# Ultrasound and magnetic resonance image findings in a patient with a subungual abscess: A case report

**DOI:** 10.1002/ccr3.8593

**Published:** 2024-03-04

**Authors:** Akihiko Nakabayashi, Atsuko Tsujii, Dong‐seop Kim, Tatsuya Tamada, Maiko Yoshimura, Kentaro Isoda, Shiro Ohshima

**Affiliations:** ^1^ Department of Rheumatology National Hospital Organization Osaka Minami Medical Center Osaka Japan; ^2^ Department of Clinical Research National Hospital Organization Osaka Minami Medical Center Osaka Japan

**Keywords:** abscess, magnetic resonance imaging, nail diseases, neoplasms, ultrasonography

## Abstract

Subungual abscesses are rare, and information about them through imaging findings is lacking. Carbon dioxide laser drainage and antibiotics are effective treatment strategies for subungual abscesses. We report a case of a 47‐year‐old male healthcare worker with a subungual abscess that improved after manual drainage alone. Ultrasound and magnetic resonance images showed a tumor (with blood flow) between the nail plate and distal phalanx. Culture tests revealed *Staphylococcus aureus*. The patient's symptoms resolved quickly and the nail returned to normal after 4 months. This is possibly the first report of a subungual abscess with ultrasound and magnetic resonance imaging findings.

## 1 INTRODUCTION

Tumors are known to occur under the nails, albeit rarely. Several studies on subungual tumors used ultrasound (US) and magnetic resonance image (MRI).^1^, ^2^, ^3^, ^4^ However, these studies seldom mention subungual abscesses. Typically, reports of subungual abscesses are only available at the case report level,^5^, ^6^, ^7^ and US and MRI images of subungual abscesses are not available to the best of our knowledge; thus, imaging findings of such abscesses do not exist. Subungual abscess treatment has been effective with carbon dioxide (CO_2_) laser drainage and antibiotic administration. We have experienced a case of a subungual abscess that improved after manual drainage without antibiotics. Here we report US and MRI images of the subungual abscess before and after treatment.

## 2 CASE HISTORY/EXAMINATION

**FIGURE 1:**
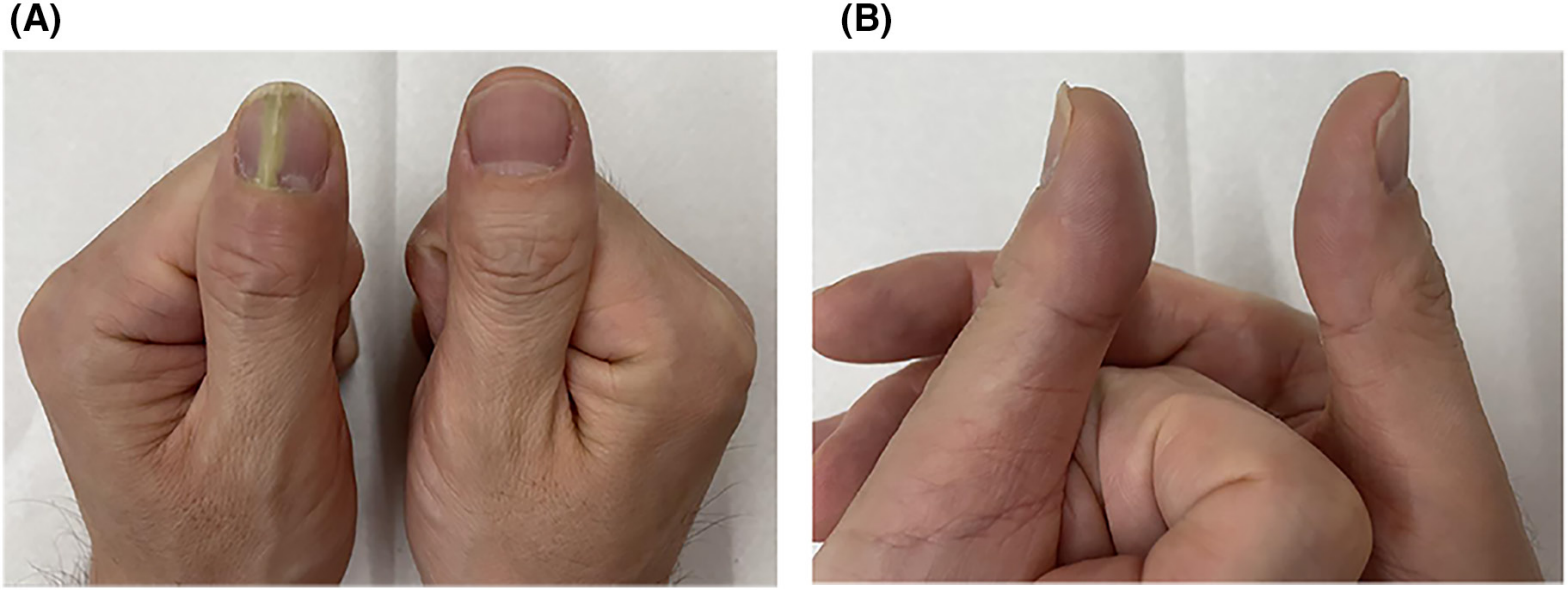
Both first fingers at the first visit. (A) The frontal view. (B) The lateral view. The nail of the left first finger has a vertical white line, the nail fold is reddish and swollen and the fingertip is enlarged compared to the right first finger.

The patient was a 47‐year‐old Japanese male healthcare worker who had no specific medical history, allergies, oral medications, or family history. He has smoked 20 cigarettes a day for the past 10 years and is only an occasional drinker. He frequently washed his hands because of the coronavirus disease‐2019 (COVID‐19) pandemic. Approximately 4 months ago, he noticed an abnormality in his left first fingernail, which had a vertical white line. He believed the nail abnormality was caused as a result of losing 10 kg body weight in an attempt to get into shape. Approximately 2 weeks ago, he gradually developed swelling, heat, and tenderness around the left first fingernail; thus, he visited the rheumatology and orthopedics department of our hospital. He had no other symptoms, that is, fever or arthralgia. His vital signs were as follows: blood pressure 135/72 mmHg, pulse 80 beats/min, respiratory rate 12 breaths/min, and SpO_2_ 99% (room air). The physical examination revealed a swollen entire left first fingertip compared with the right first fingertip and a reddish proximal nail fold with evidence of paronychia. In addition, he had tenderness when pressing on the proximal nail fold and the proximal end of the nail plate (Figure 1).

## 3 DIFFERENTIAL DIAGNOSIS, INVESTIGATIONS, AND TREATMENT

**FIGURE 2:**
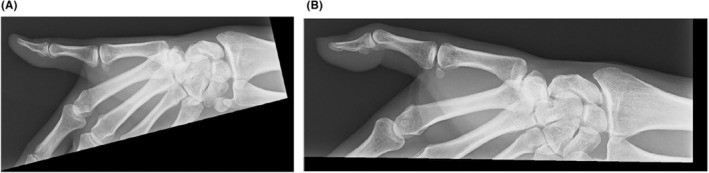
Radiographic findings of both first fingers at the first visit. (A) The right first finger on X‐ray. (B) The left first finger on X‐ray. No fracture or bone erosion is seen in the left first finger. The left first finger has more soft tissue swelling than the right first finger.

**FIGURE 3:**
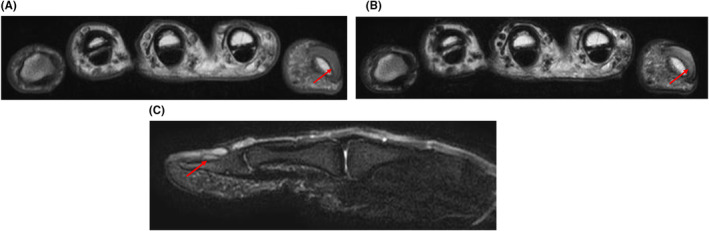
MRI findings of left fingers at the first visit. (A) The left fingers on the T1‐weighted axial image, (B) the left fingers on the T2‐weighted axial image, and (C) the left first finger on STIR sagittal image. MRI showed a tumor with low intensity on T1‐weighted axial image and high intensity on T2‐weighted axial image and the short‐tau inversion recovery (STIR) sagittal image between the nail plate and the distal phalanx (red arrow). The left first fingernail and the tumor are not in contrast and the border is indistinct on the T1‐weighted image. The left first fingernail is thick and high intensity, unlike the second through fifth fingernail on the T2‐weighted image. MRI: magnetic resonance image. STIR, short‐tau inversion recovery.

**FIGURE 4:**
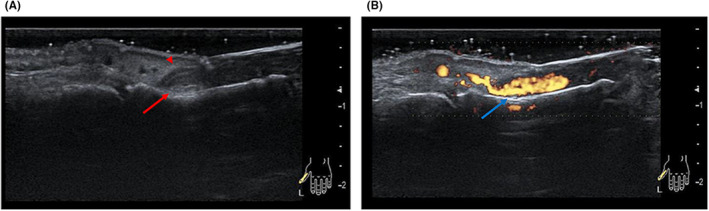
US sagittal scan of the left first finger at the first visit. (A) Greyscale, (B) Power Doppler (see also Videos S1). The US revealed an oval and well‐defined tumor between the nail plate and the distal phalanx (red arrow). The tumor was pushing up the nail root (red arrowhead). The interior of the tumor was isoechoic with abundant blood flow within the tumor (blue arrow). US, ultrasound.

**FIGURE 5:**
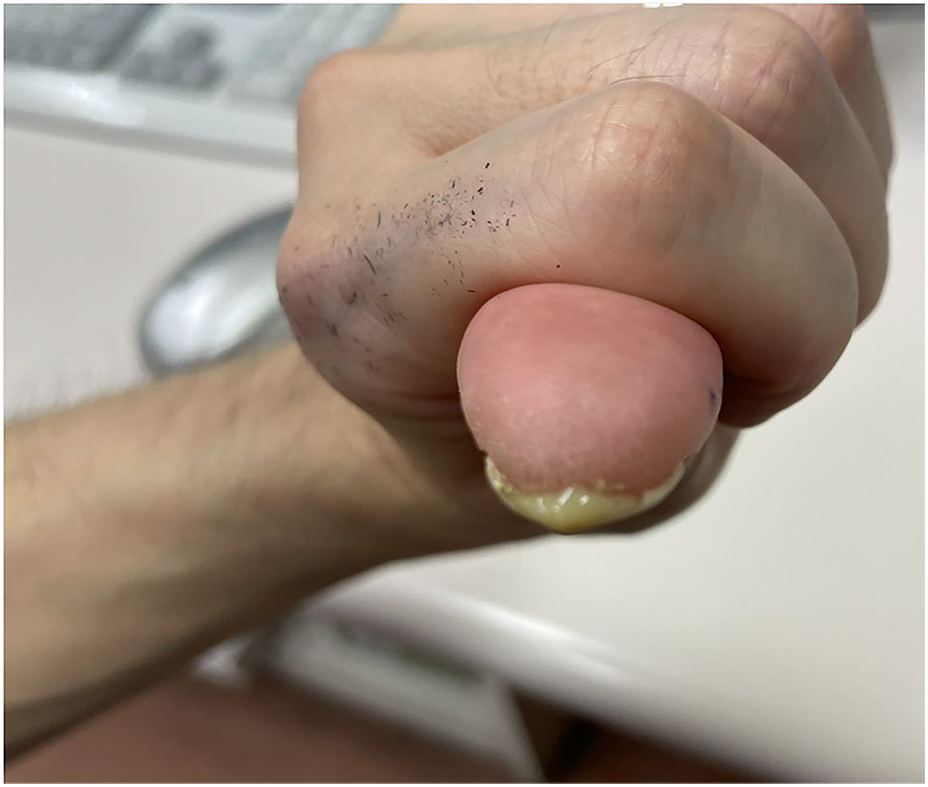
The pus from the onychodermal band. A culture examination revealed *S. aureus*. S. aureus, Staphylococcus aureus.

**FIGURE 6:**
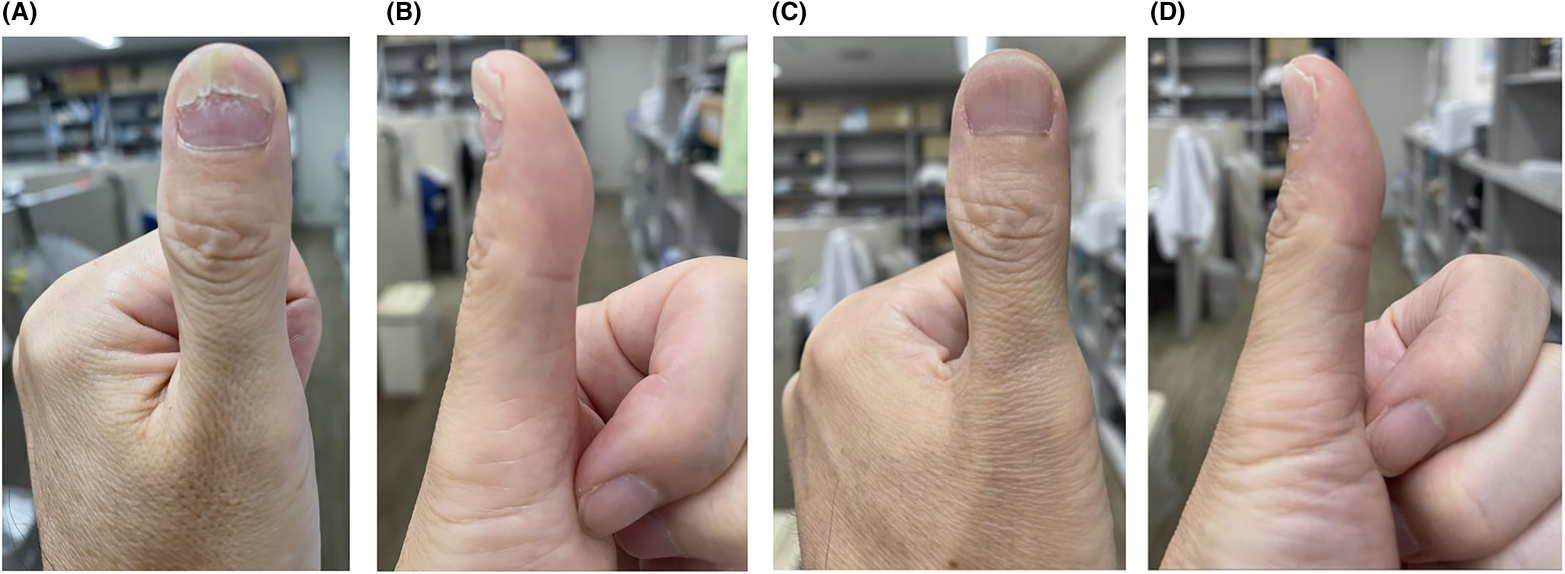
Improvement of the left first finger over time. (A) The frontal view after 2 months. (B) The lateral view after 2 months. (C) The frontal view after 4 months. (D) The lateral view after 4 months. The left fingernail gradually grew back to normal, and it completely improved after 4 months.

**FIGURE 7:**
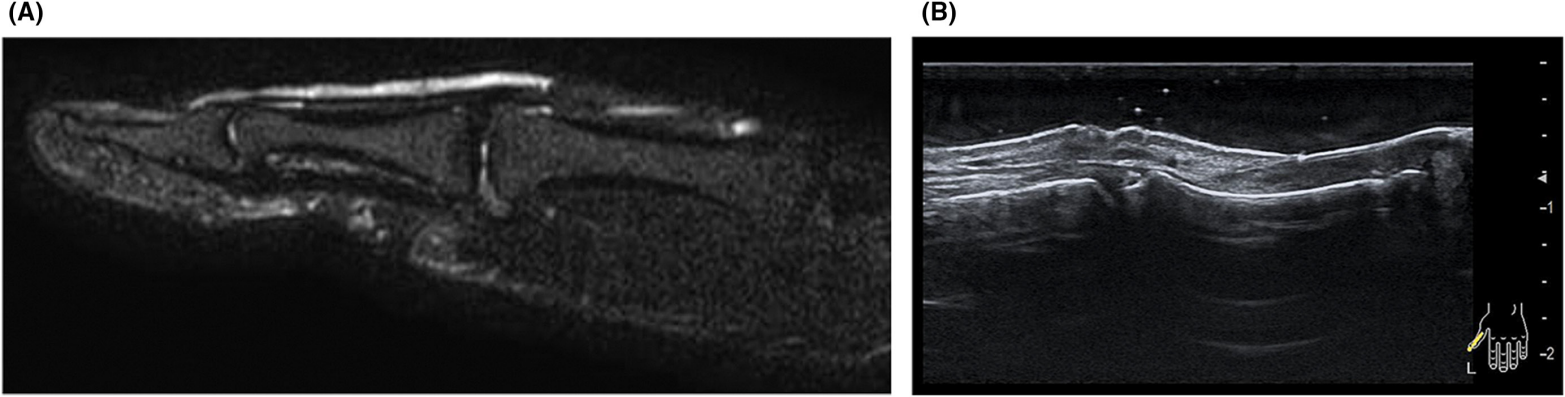
MRI and US findings after 4 months. (A) The left first finger on STIR sagittal image, (B) the left first finger on US sagittal scan (see also Videos S2). The subungual abscess had completely disappeared. MRI: magnetic resonance image. US, ultrasound. STIR, short‐tau inversion recovery.

The X‐ray revealed no fractures or bone erosion; however, soft tissue swelling was observed in the vicinity of the nail and at the palmar distal pulp (Figure 2). We performed MRI of the left fingers using a 3 Tesla scanner without contrast media (Ingenia 3.0 T, Philips Healthcare, Best, the Netherlands). MRI showed a tumor with low intensity on T1‐weighted axial image and high intensity on T2‐weighted axial image and the short‐tau inversion recovery (STIR) sagittal image between the nail plate and the distal phalanx (Figure 3). The tumor had a relatively long extension both vertically and horizontally. A rheumatologist with 8 years of experience performed a high‐frequency US examination using the ARIETTA 850 device (FUJIFILM Healthcare Corporation, Tokyo, Japan) with a 2–22 MHz transducer. The US image revealed an oval and well‐defined tumor between the nail plate and the distal phalanx. The tumor was pushing up the nail root. The interior of the tumor was isoechoic with abundant blood flow within the tumor (Figure 4, Videos S1). The patient then pressed the proximal nail fold and found pus from the onychodermal band (Figure 5). Culture examination revealed a beta‐lactamase‐producing form of *Staphylococcus aureus* (*S. aureus*), which was resistant to penicillin G and aminobenzylpenicillin but suitably sensitive to other antibiotics. Manual pressing of the proximal and bilateral nail folds resulted in adequate drainage and immediate improvement of symptoms without antibiotics, and the nail was completely improved in 4 months (Figure 6). At the same time, the nail improved, and MRI and US showed a complete resolution of the subungual abscess (Figure 7; Videos S2).

## 4 OUTCOME AND FOLLOW‐UP

Therefore, a final diagnosis for the subungual abscess was established. One year and 7 months have passed since then, and no recurrence has been observed.

## 5 DISCUSSION

We experienced a case of subungual abscess with paronychia that improved with manual drainage without antibiotics. Among the numerous reviews on subungual tumors using US and MRI,^1^, ^2^, ^3^, ^4^ the most comprehensive is that of Mundada et al.^1^ This review broadly divided subungual tumors into nail tumors and nail tumor‐like lesions (e.g., mucoid cyst and epidermoid cyst). Nail tumors are divided into benign (e.g., glomus tumors and subungual exostosis, etc.) and malignant tumors (e.g., squamous cell carcinoma and malignant melanoma). They also stated that high‐resolution MRI can identify subungual tumors better than X‐ray or US although not in a perfect manner. However, four studies, including this one, did not include subungual abscesses. Subungual abscesses are mainly reported in case reports,^5^, ^6^, ^7^ and no reports included US or MRI images as far as we have examined. All these three cases were improved through CO_2_ laser drainage of the abscess and antibiotic treatment; however, the current case was improved through manual drainage without antibiotics. No studies have reported the organisms responsible for subungual abscesses; however, a study reported the organisms responsible for paronychia, with the *Enterobacteriaceae family* being the most common, followed by the *Staphylococci genus*.^8^ The causative organism in our case was *S. aureus*, which is the most common member of the *S. genus*. Paronychia has been reported to be more common in occupations that involve frequent handling of water, such as bartenders, housekeepers, housemakers, dishwashers, and swimmers.^8^ The patient in the present case was a healthcare worker who frequently washed his hands because of the COVID‐19 pandemic, which may have caused the subungual abscess. Anticancer agents, such as taxanes and anthracyclines, have also been reported as a cause of subungual abscesses, but no drugs were used in the present case.^9^

Ávila de Almeida et al. reported one subungual tumor, a glomus tumor, using a 33 MHz transducer with very sophisticated US images while previous US studies of subungual tumors were reported using a 15–18 MHz transducer.^10^ We clearly delineated a subungual abscess before and after treatment using a high‐frequency transducer of 2–22 MHz in the present case, similar to that reported by Ávila de Almeida et al.

Abscesses should also be listed in the differential diagnosis of subungual tumors, and this is the first report of a subungual abscess that included US and MRI images.

## AUTHOR CONTRIBUTIONS

**Akihiko Nakabayashi:** Conceptualization; data curation; formal analysis; funding acquisition; investigation; methodology; project administration; resources; software; supervision; validation; visualization; writing – original draft; writing – review and editing. **Atsuko Tsujii:** Writing – review and editing. **Dong‐seop Kim:** Writing – review and editing. **Tatsuya Tamada:** Writing – review and editing. **Maiko Yoshimura:** Writing – review and editing. **Kentaro Isoda:** Writing – review and editing. **Shiro Oshima:** Supervision.

## FUNDING INFORMATION

No specific funding was received to conduct the work described in this manuscript.

## CONFLICT OF INTEREST STATEMENT

The authors declare that they have no competing interests.

## ETHICS STATEMENT

This study has not been approved by the hospital committee to report this case report. However, the person in charge of the hospital verbally agreed. Written consent from the patient was obtained for participation.

## CONSENT

Written informed consent was obtained from the patient for publication of this case report and any accompanying images.

## Supporting information


Video S1.

Video S2.

## Data Availability

None.

## References

[1] Mundada P , Becker M , Lenoir V , et al. High resolution MRI of nail tumors and tumor‐like conditions. Eur J Radiol. 2019;112:93‐105. doi:10.1016/j.ejrad.2019.01.004 30777226

[2] Baek HJ , Lee SJ , Cho KH , et al. Subungual tumors: clinicopathologic correlation with US and MR imaging findings. Radiographics. 2010;30:1621‐1636. doi:10.1148/rg.306105514 21071379

[3] Singh R , Bryson D , Singh HP , Jeyapalan K , Dias JJ . High‐resolution ultrasonography in assessment of nail‐related disorders. Skeletal Radiol. 2012;41:1251‐1261. doi:10.1007/s00256-012-1426-1 22609988

[4] Aluja Jaramillo F , Quiasúa Mejía DC , Martínez Ordúz HM , González AC . Nail unit ultrasound: a complete guide of the nail diseases. J Ultrasound. 2017;20:181‐192. doi:10.1007/s40477-017-0253-6 28900518 PMC5573700

[5] Patel S , Lloyd JR . Subungual abscess caused by staphylococcus lugdunensis. Cutis. 2013;92:125‐126. 24153139

[6] Fleming TE , Brodell RT . Subungual abscess: a bacterial infection of the nail bed. J Am Acad Dermatol. 1997;37:486‐487. doi:10.1016/s0190-9622(97)70153-1 9308567

[7] Lee H , Mun JH . Subungual abscess treated by decompression using a CO_2_ laser. Indian J Dermatol Venereol Leprol. 2020;86:449‐451. doi:10.4103/ijdvl.IJDVL_392_19 32415049

[8] Tomczak H , Dańczak‐Pazdrowska A , Polańska A , et al. Microbiological analysis of acute infections of the nail fold on the basis of bait thread test. Postepy Dermatol Alergol. 2017;34:110‐115. doi:10.5114/ada.2017.67072 28507488 PMC5420601

[9] Piraccini BM , Alessandrini A . Drug‐related nail disease. Clin Dermatol. 2013;31:618‐626. doi:10.1016/j.clindermatol.2013.06.013 24079591

[10] Ávila de Almeida C , Guarçoni S , Leverone A , Nakamura R , Marchiori E , Canella C . Characterization of a glomus tumor using 33‐MHz ultrasound and superb microvascular imaging. Skin Res Technol. 2021;27:466‐468. doi:10.1111/srt.12972 33141970

