# Supplementary material for: Ultrasound and magnetic resonance image findings in a patient with a subungual abscess: A case report
Source: Clin Case Rep. 2024 Mar 4;12(3):e8593. doi: 10.1002/ccr3.8593 (PMC10912096; doi:10.1002/ccr3.8593)
Supplement: Supplementary file 1 — Video S1. [file CCR3-12-e8593-s002.docx]

Supplementary Video 1: US sagittal scan of the left first finger at the first visit. (a) Greyscale, (b) Power Doppler.

Supplementary Video 2: US sagittal scan of the left first finger after 4 months.
