# Supplementary figures and images for: Ultrasound and magnetic resonance image findings in a patient with a subungual abscess: A case report
Source: Clin Case Rep. 2024 Mar 4;12(3):e8593. doi: 10.1002/ccr3.8593 (PMC10912096; doi:10.1002/ccr3.8593)

## Slide 1
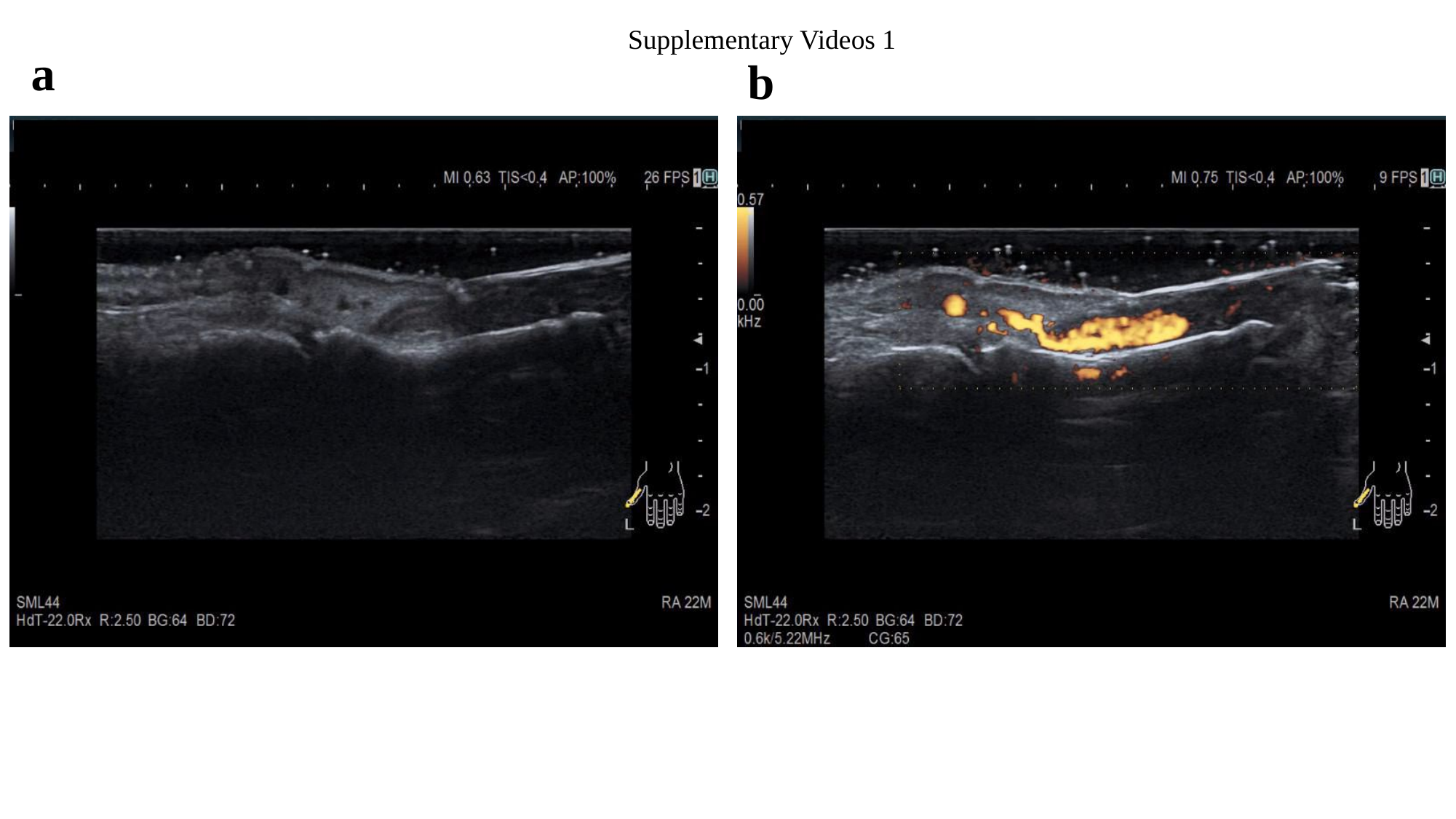

Supplementary Videos 1
a
b

## Slide 2
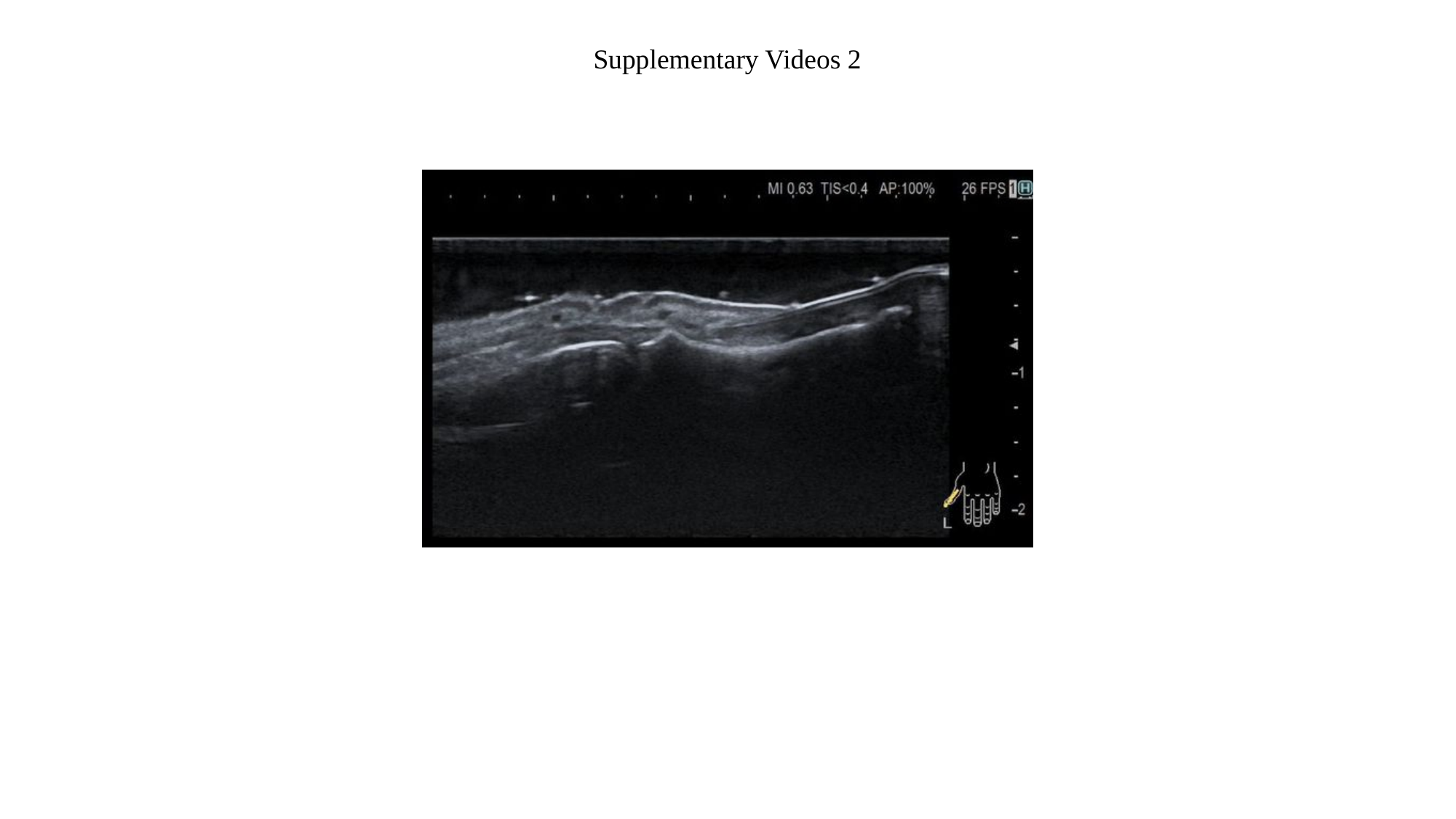

Supplementary Videos 2

Supplement: Supplementary file 2 — Video S2. [file CCR3-12-e8593-s001.pptx]
